# The Real-Time Validation of the Effectiveness of Third-Generation Hyperbranched Poly(ɛ-lysine) Dendrons-Modified KLVFF Sequences to Bind Amyloid-β_1-42_ Peptides Using an Optical Waveguide Light-Mode Spectroscopy System

**DOI:** 10.3390/s22239561

**Published:** 2022-12-06

**Authors:** Valeria Perugini, Matteo Santin

**Affiliations:** Centre for Regenerative Medicine and Devices, School of Applied Sciences, University of Brighton, Huxley Building Lewes Road, Brighton BN2 4GJ, UK

**Keywords:** Alzheimer’s disease, in vitro diagnostics, Aβ_1-42_ amyloids, Aβ_1-42_ fibril inhibitors, KLVFF, poly(epsilon-Lysine) dendrons, optical waveguide lightmode spectroscopy

## Abstract

The aggregation of cytotoxic amyloid peptides (Aβ_1-42_) is widely recognised as the cause of brain tissue degeneration in Alzheimer’s disease (AD). Indeed, evidence indicates that the deposition of cytotoxic Aβ_1-42_ plaques formed through the gradual aggregation of Aβ_1-42_ monomers into fibrils determines the onset of AD. Thus, distinct Aβ_1-42_ inhibitors have been developed, and only recently, the use of short linear peptides has shown promising results by either preventing or reversing the process of Aβ_1-42_ aggregation. Among them, the KLVFF peptide sequence, which interacts with the hydrophobic region of Aβ_16-20_, has received widespread attention due to its ability to inhibit fibril formation of full-length Aβ_1-42_. In this study, hyperbranched poly-L-lysine dendrons presenting sixteen KLVFF at their uppermost molecular branches were designed with the aim of providing the KLVFF sequence with a molecular scaffold able to increase its stability and of improving Aβ_1-42_ fibril formation inhibitory effect. These high-purity branched KLVFF were used to functionalise the surface of the metal oxide chip of the optical waveguide lightmode spectroscopy sensor showing the more specific, accurate and rapid measurement of Aβ_1-42_ than that detected by linear KLVFF peptides.

## 1. Introduction

Alzheimer’s disease (AD) is a neurodegenerative condition for which no effective medical treatment to either reverse or significantly retard the pathological progression is currently available [1]. However, insights into the biological mechanisms underlying AD have been emerging, particularly in relation to the identification of specific biochemical pathways of disease onset and progression [2]. Among them, the progressive aggregation of amyloid β_1-42_ peptides (Aβ_1-42_) into fibrils and cytotoxic plaques is now considered the main cause of the disease [3,4]. Independent of their length, localisation and amino acid composition, these aggregates induce synaptic dysfunction and neuronal death [4].

This ascertained role of Aβ_1-42_ amyloids in AD has encouraged their use as biomarkers for the early diagnosis and monitoring of disease progression alongside cognitive tests [5]. Unlike the other accepted biomarkers, the tau and phosphorylated tau protein, Aβ_1-42_ amyloids are able to indicate the onset of the disease before neural cytotoxicity takes place. Studies have shown compelling evidence that the combined analysis of amyloids and tau biomarkers in the cerebrospinal fluid (CSF) may predict the development of AD in patients who already show mild cognitive impairment. However, the available enzyme-linked immuno-assays (ELISA) kits used for their detection are time-consuming and not sufficiently sensitive to measure small decreases in concentration occurring in the CSF and are even less reliable if applied to the analysis of blood samples where the concentrations of this biomarker are relatively lower [6].

Short peptides able to prevent Aβ_1-42_ aggregation have been proposed as potential therapeutic agents because of their binding affinity to specific regions of the Aβ_1-42_ [7,8,9,10,11,12]. Among the wide range of Aβ_1-42_ aggregation peptide blockers, only those incorporating the amino acid sequence KLVFF have been demonstrated to selectively prevent amyloid aggregation [13,14,15,16]. By specifically binding to the homologous regions of Aβ_1-42_, KLVFF aggregation blockers induce the formation of an atypical anti-parallel β-sheet structure primarily distributed at their hydrophobic residues (e.g., KLF) [17]. Despite these promising results, both insolubility and toxicity issues have prevented KLVFF aggregation blockers from reaching their final clinical and therapeutic use [11]. Furthermore, their potential as molecules able to detect specifically and sensitively Aβ_1-42_ in CSF and blood remains largely unexplored [10].

Recently, hyperbranched poly(epsilon-Lysine) peptides have been developed as molecules for the functionalisation of material surfaces ranging from metal to polymer where they have been shown to be able to improve biointeractions by presenting specific bioligands through high-density and controlled molecular spacing [11,12,13]. However, their potential to increase the diagnostic potential of biosensors and point-of-care in vitro diagnostics has not yet been considered [14].

The present work shows the integration of the KLVFF sequence at the uppermost molecular branching of poly(ɛ-lysine) dendrons of 3 branching generations (i.e., 16 uppermost molecular branches), demonstrating their increased binding affinity to Aβ_1-42_ peptides and enhanced capacity as aggregation blockers when compared with the linear KLVFF peptide. The branched molecular design of the Aβ_1-42_ aptamer was then harnessed as a biospecific surface functionalisation moiety of optical waveguide lightmode spectroscopy (OWLS) chip sensor surfaces. The OWLS sensing principle relies upon the grating of the titanium oxide surface of the chip incoupling light into a planar optical waveguide through which the light propagates, generating an evanescent field [15,16,17,18]. The range of this field extends slightly above the grating (approximately 100 nm), allowing it to probe the composition of a water sample flowing over the sensor. The presence of bound material on the sensor leads to a change in the refraction index of the covering medium, which is detected by two photoelectric sensors set perpendicular to the laser beam at opposite sides of the waveguide and is measured as transverse electric and magnetic currents. This enables the monitoring of a change in the position of the transverse electric and transverse magnetic peaks as they shift to higher values as interactions occur.

This paper aims to evaluate the binding properties of linear and dendron-presented KLVFF aptamers and their potential as a surface functionalisation method of OWLS chips for the real-time and sensitive detection of Aβ_1-42_ amyloid precursors.

## 2. Materials and Methods

### 2.1. Hyperbranched KLVFF Design

**Figure 1:**
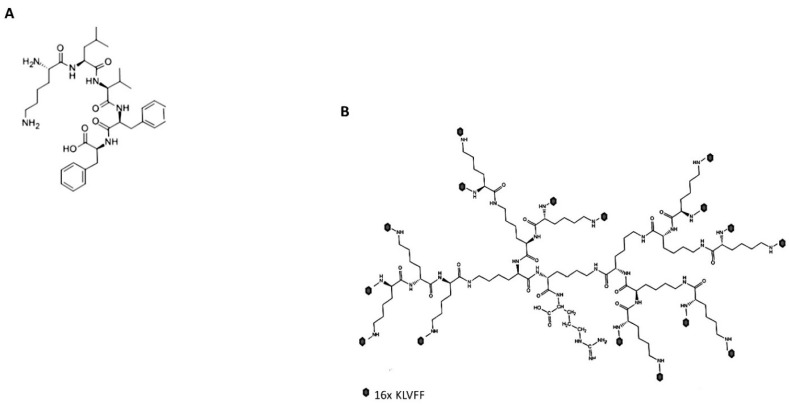
Molecular structures of the linear KLVFF (**A**) and hyperbranched KLVFF [Rgen3K(KLVFF)_16_] (**B**). Dark dots at the terminal of the Rgen3K(KLVFF)_16_ branching indicate the KLVFF sequence.

The pentapeptide KLVFF sequence identified by Tjernberg [19] was selected because of its reported high affinity for Aβ_1-42_ peptides [19] and because of its N-terminal (lysine) and carboxy-terminal (di-phenylalanine) that were favourable to the integration into the poly(epsilon-Lysine) dendron, respectively (Figure 1A). The poly (epsilon-Lysine) dendron was designed as three generations of branching (16 uppermost molecular branches) to present the KLVFF sequence at high density to the Aβ_1-42_ peptides, while minimising any steric hindrance that could potentially reduce the quality of the synthesis. The molecular root of these dendrons was designed to be based on a monomer of arginine (R) that could strengthen the interaction with the negative surface of the sensor chip based on a negatively charged metal oxide surface while repelling the remaining positively charged core of the hyperbranched peptide thus reducing the risk of its collapsing on the sensor surface and maximise its exposure to the surrounding liquid environment (Figure 1B).

### 2.2. Synthesis and Characterisation of Linear and Hyperbranched KLVFF

The assembly of the linear KLVFF and hyperbranched dendron-presented KLVFF [Rgen3K(KLVFF)_16_] was performed using a common 9-fluorenylmethoxycarbonyl (Fmoc) solid phase peptide method by a microwave synthesiser (Biotage, Hengoed, UK). Initially, 0.5 g of Tenta gel resin was swollen in N, N –dimethylformamide (DMF) (Fisher Scientific, Loughborough, UK) for 15 min at room temperature. After three washes with 3 cm^3^ of DMF, the resin was coupled to the carboxy groups of rink amide linker (Iris Biotech GmbH, Marktredwitz, Germany) that was previously sonicated with 3 cm^3^ of DMF and 0.4 mmol M O-Benzotriazole-N,N,N’,N’-tetramethyl-uronium-hexafluoro-phosphate (HBTU) and 0.8 mmol *v*/*v* N,N-diisopropylethylamine (DIPEA) (Sigma Aldrich Co. Ltd., Cambridge, UK). The mixture was allowed to react at 50 °C, 900 rpm and high absorption for 5 min. This was then rinsed three times with 3 cm^3^ of DMF and treated twice with 20% *v*/*v* piperidine in DMF (Sigma Aldrich Co. Ltd., Cambridge, UK) at room temperature for 4 min to allow the Fmoc-groups of the linker to be removed while allowing a new N-terminal amine to be revealed and used for supporting the assembly of each peptide chain.

After the synthesis, all peptides were cleaved from resin using a cocktail of 95% *v*/*v* of TFA, trisopropylsilane (TIPS) and deionised H_2_O (2.5% *v*/*v*). These were incubated for three hours and then collected in chilled diethylether (Fisher Scientific, Loughborough, UK) before being washed three times at 3500 rpm for 5 min. Afterwards, peptides were lyophilised using a Christ Alpha2-4 freeze-dryer overnight and characterised using mass spectroscopy (Bruker microTOF, Coventry, UK) and HPLC (Agilent Infinity, Palo Alto, CA, USA).

Fourier transform infrared spectroscopy (FTIR) was carried out using a Perkin Elmer Spectrum 65. Infrared spectra were measured over 16 scans at a frequency range of 4000–550 cm^−1^ and resolution limit of 4 cm^−1^. HPLC and mass spectrometry were also performed to analyse the purity of the final product as its identification as compared to the theoretical molecular weight. HPLC method (WatersTM 717 plus autosampler, Waters, Wilmslow, UK) performed on a hydrophobic RP 18 column (150 × 4.60 mm, Luna 3u C18 100A, Phenomenex) at 25 °C (Column chiller Model 7955, Jones Chromatography, Hengoed, UK). The mobile phase consisted of a stepwise gradient of solvent A 1% *v*/*v* TFA in deionized water and solvent B 0.1% *v*/*v* TFA in acetonitrile. Chromatograms were recorded on UV detector (SPO-6A, Shimadzu, Tokyo, Japan) and analyzed by HPLC software, Total Chrom-TC Navigator. Mass spectrometry was performed by microTOF (Bruker, Coventry, UK).

An additional previously established scrambled peptide sequence (Rgen3K(VFLKF)_16_) was also synthesised using the same procedure and utilised as a negative control in the Aβ_1-42_ self-aggregation experiments.

### 2.3. Thioflavin T Staining

The ability of both linear and branched KLVFF to prevent Aβ_1-42_ fibril self-assembly were initially assessed with thioflavin T (ThT). Briefly, 10 µM Aβ fibrils were mixed with and without KLVFF peptides and added into a glass beaker filled up with silicon oil that it is known to induce amyloid aggregation and therefore able to demonstrate any aggregation inhibitory effect caused by the presence of the blockers. The inhibitor concentrations were set at the same molar concentration (50 µM) of the active principle (KLVFF) rather than of the whole molecules (linear vs. branched KLVFF). The solutions were then left in incubation at 50 °C under continuous agitation (70 rpm) to maximise aggregation conditions and upon a period of 7 days to monitor the effect of the linear and branched blockers upon time. Afterwards, individual samples were mixed with 10 µL of a ThT solution that was previously prepared in deionized water (5 µM) and filtered through a syringe filter with a pore diameter of 0.25 µm (GE Healthcare, Amersham, UK). After 30 min incubation at 37 °C, 150 µL, each solution was added onto tissue culture plate well (Nunc, Rochester, NY, USA) for 10 min at room temperature before being examined by a fluorescence microscopy (Nikon Elipse TE2000U, UK) using a fluorescent dye at the excitation wavelength of 350 nm and an emission wavelength of 438 nm.

### 2.4. Congo Red Assay

Samples of Aβ_1-42_ with and without the linear and branched KLVFF were prepared in the same way of ThT assay. After 1 day and 7 days’ incubation, samples were stained with 25 µM of Congo red for 20 min and left in alkaline alcohol solution before being rinsed three times with water. The absorbance from each sample was then quantified using a spectrophotometer plate reader (Biotek, Stockport, UK) at 540 (A_540_) and 480 nm (A_480_).

### 2.5. Optical Waveguide Lightmode Spectroscopy Chip Surface Functionalisation

The coupling of both linear and branched KLVFF was monitored by an optical waveguide lightmode spectroscopy (OWLS, Microvacuum Ltd., Budapest, Hungary) using a method previously established. Briefly, an amino-functionalised OWLS sensors (OW2400) containing an optical titanium grating (2 × 16 mm × 20 nm) was placed on the top of a glass support and inserted into a specific OWLS holder. The waveguide sensor surface was initially washed with deionised water for around 20 min and then the hydroxyl groups were functionalised with three injections of 2.5% *w*/*v* glutaraldehyde (50 μL Sigma Aldrich, Cambridge, UK) at a flow rate of 0.20 mL min^−1^ at 25 °C. Afterwards, a stable baseline was achieved by flowing deionised water through the chip prior to the injection of either linear or branched KLVFF (50 μL) at a final concentration of 10 mg/mL. Real-time binding of the two types of KLVFF molecules and elution of the unbound excess were monitored until a stable plateau was reached.

### 2.6. OWLS Study of Aβ_1-42_ Binding

Fibrillar Aβ_1-42_ preparation. Protein Aβ_1-42_ fragments (Sigma Aldrich, Cambridge, UK) were initially diluted in DMSO (5 mM) at room temperature and then added to 10 mM HCl in order to obtain a final concentration of 100 µM. The solution was quickly vortexed and incubated to 37 °C for 48 h.

The addition of 50 μL Aβ_1-42_ (5 mg/mL, Sigma Aldrich, Cambridge, UK) onto the OWLS chip followed its functionalisation with the KLVFF or Rgen3K(KLVFF)_16_ where flow rates were maintained at 0.20 mL min**^−^**^1^ during adsorption and elution steps. Aβ_1-42_ was also injected on bare OWLS chips and used as controls (*n* = 2).

All measurements were analysed by the MicroVacuum BioSense Software (MicroVacuum Ltd., Budapest, Hungary) which calculated (i) the intensity peak angles for both the transverse electric (IntTE) and the transverse magnetic (IntTM) of the absorbed linear and branched KLVFF peptides on the waveguide sensor surfaces and (ii) their respective mass (M) using the Feijter equation (Equation (1)):

*M = d_A_ × (n_A_ − n_C_)* *(d_n_/d_c_)* (1)

where *d_A_* = thickness of the added layer, *n_A_* = refractive index of the analyte layer, *n_C_* = refractive index of the cover medium and refractive index increment *d_n_*/*d_c_* = 0.182 cm^3^/g.

### 2.7. Statistical Analysis

Statistical analysis was performed ANOVA on *n* = 3 and values were considered significantly different at *p* ≤ 0.05.

## 3. Results

### 3.1. Characterisation of Linear KLVFF and Rgen3K(KLVFF)_16_ Peptides

#### Physicochemical Characterisation

**Figure 2:**
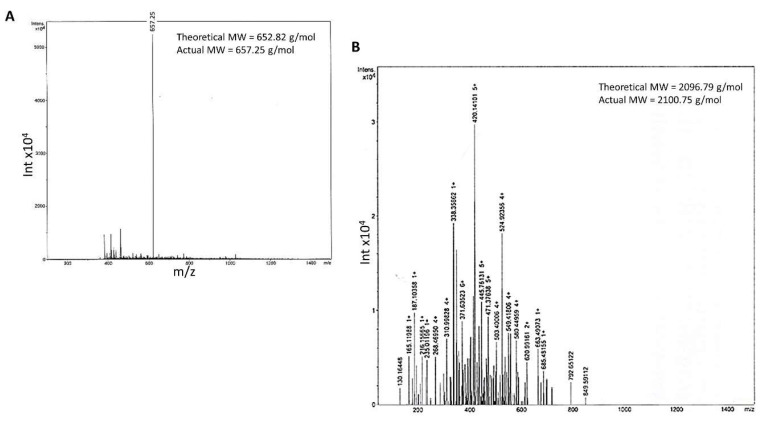
Mass spectrometry of the linear KLVFF (**A**) and Rgen3K (**B**). Theoretical and experimental molecular weights are reported in the spectra (Rgen3K(KLVFF)_16_ spectrum data not shown as its molecular weight was outside the detection range of the equipment).

**Figure 3:**
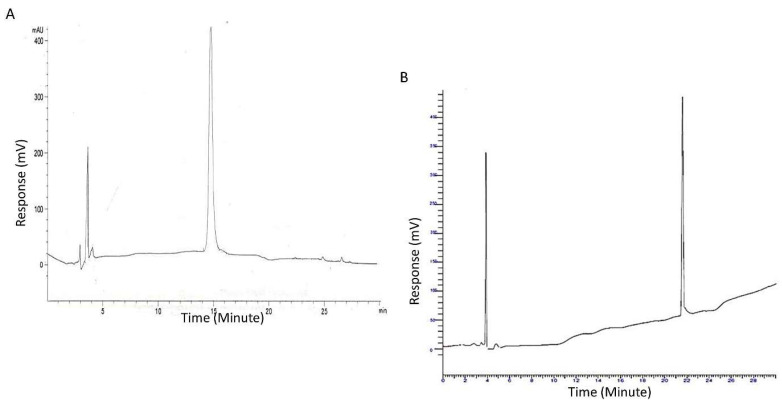
HPLC chromatograms of the linear KLVFF (**A**) and Rgen3K(KLVFF)_16_ (**B**). Peak at 4 min is assigned to the organic solvent used as mobile phase of the chromatography.

- Both linear and branched KLVFF peptides (Figure 1A,B) were assembled in batches of approximately 80 mg and successfully characterised by mass spectrometry (Figure 2A,B) and HPLC (Figure 3). Their degree of purity was always above 95% with yields of reaction 75 and 81% for KLVFF and Rgen3K(KLVFF)_16_, respectively.

**Figure 4:**
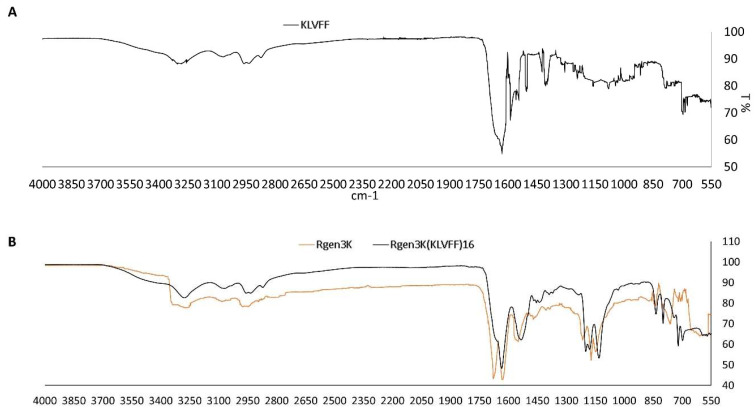
FTIR of linear KKVFF (**A**), Rgen3K and Rgen3K(KLVFF)_16_ (**B**).

The comparison of the Rgen3K(KLVFF)_16_ FTIR spectrum with those of the linear KLVFF (Figure 4A) and of the control Rgen3K shows the presence of the KLVFF peaks, particularly those in the 650 to 1200 cm^−1^ region, within the hyperbranched Aβ_1-42_ blocker (Figure 4B). Together with the presence of a single HPLC peak, this analysis result demonstrates the integration of the linear sequence within the hyperbranched structure.

### 3.2. Biospecific Binding Tests and OWLS Sensing of Aβ_1-42_

#### 3.2.1. Linear and Branched KLVFF Peptides Blocking Effect on Aβ_1-42_ Fibrils Formation

**Figure 5:**
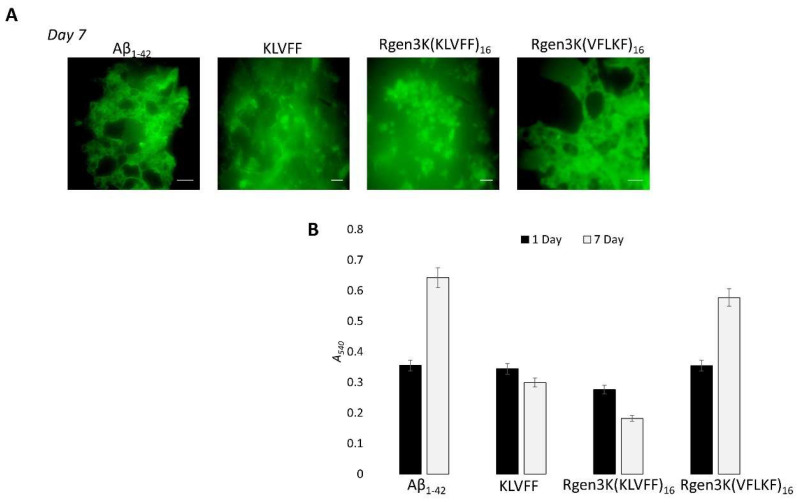
Inhibitory effect of Aβ_1-42_ fibril formation by linear KLVFF and hyperbranched Rgen3K(KLVFF)_16_ assessed by confocal microscopy of ThT-stained (**A**) and Congo Red-stained ((**B**) Rgen3K(KLVFF)_16_) samples over 7 days’ incubation. Effective binding specificity was proven using a scrambled hyperbranched sequence as control. Scale bar in A: 50 nm. Error bars in B indicate standard deviations. Statistically significant differences at *p* ≤ 0.05 were observed in the case of samples treated with KLVFF and Rgen3K(KLVFF)_16_ peptides.

- To monitor the potential of both peptides to inhibit the self-aggregation of Aβ_1-42_ monomers into fibrils as well as the disruption of formed fibrils, ThT and Congo red analysis were performed and used to quantify the amounts of formed fibrils when Aβ_1-42_ was incubated with either linear or branched KLVFF peptides for 1 and 7 days (Figure 5A,B). Both fluorescent microscopy of ThT-stained samples (Figure 5A) and spectrophotometric measurement of Congo Red staining (Figure 5B) clearly show the enhanced inhibition of Aβ_1-42_ peptide aggregation when incubated with hyperbranched Rgen3K(KLVFF)_16_. The specificity of the binding was also demonstrated when a scramble sequence (i.e., VFLKF) of the peptide was tested showing no disaggregation properties.

#### 3.2.2. OWLS Real-Time Monitoring of Aβ_1-42_

**Figure 6:**
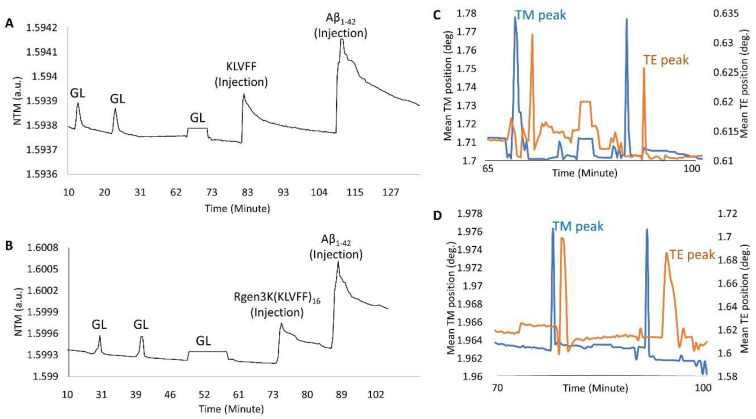
OWLS measurements of the linear KLVFF (**A**,**C**) and hyperbranched Rgen3K(KLVFF)_16_ (**B,D**) followed by their relative Aβ_1-42_ binding. (**A**,**B**) show the activation of the OWLS chip metal oxide surface with glutaraldehyde (GL) followed by washing steps and grafting of either KLVFF ((**A**), KLVFF injection) or Rgen3K(KLVFF)_16_ ((**B**), Rgen3K(KLVFF)_16_ injection). (**C**,**D**) show the respective intensity peak angles for both the transverse electric (IntTE) and the transverse magnetic (IntTM). Third GL injection shows formation of a plateau indicating surface saturation. Change in medium after the third GL injection leads to a slight change in the baseline signal. Experiments also include the following injection of the Aβ_1-42_ samples.

**Figure 7:**
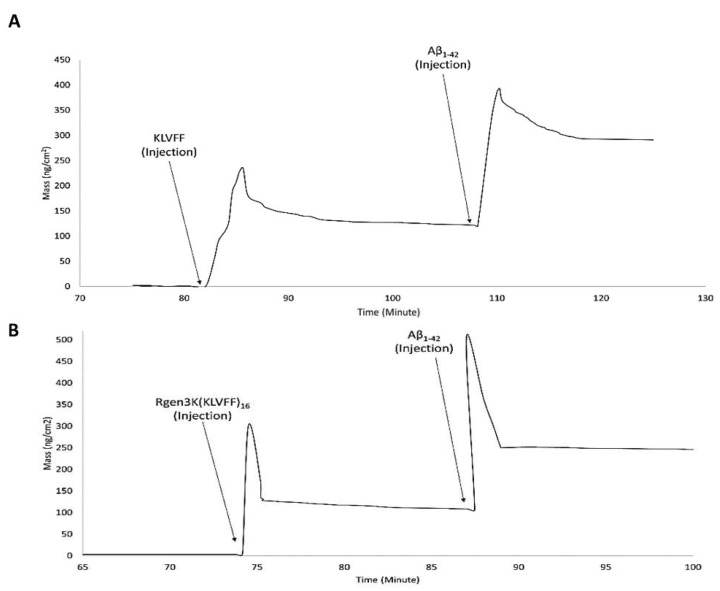
OWLS real-time monitoring of binding mass of functionalisation molecules and Aβ_1-42_ monomers. OWLS chip functionalised with linear KLVFF (KLVFF injection) (**A**) and Rgen3K(KLVFF)_16_ (Rgen3K(KLVFF)_16_ injection) (**B**).

- OWLS studies clearly demonstrated the ability of both KLVFF peptides to form macromolecular complexes with Aβ_1-42_ when used to functionalise the surface of the waveguide sensor (Figure 6A,B). The binding of the linear and hyperbranched peptides to the metal-oxide thin layer of the sensor was obtained by three injections of glutaraldehyde up to allege saturation of the whole surface (Figure 6A,B,G,L); this surface derivatisation step allowed each peptide to be effectively grafted onto the sensor chip surface. However, a slightly weaker immobilisation was recorded in the case of the linear KLVFF peptides (N_TM_ = 1.5939 v N_TM_ = 1.5999). The data were also confirmed by their respective InTM (absorption phase, KLVFF = 1.78°; Rgen3K(KLVFF)_16_ = 1.97°) and InTE (desorption phase, KLVFF = 0.63°; Rgen3K(KLVFF)_16_ = 1.7°) peaks (Figure 6C,D) whereby the introduction of a water solution and indeed the stabilisation of a baseline was reached after 32 min by KLVFF rather than 15 min as observed for Rgen3K(KLVFF)_16_. This in turn directly raised the thicknesses of the coating layer from 0.01 to 2.51 [KLVFF] and 3.41 nm [Rgen3K(KLVFF)_16_] and induced the OWLS signals to significantly change within few minutes after the injections of Aβ_1-42_ [KLVFF N_TM_ = 1.5941; Rgen3K(KLVFF)_16_ N_TM_ = 1.6007].

- NTM peaks also showed the successful binding of Aβ_1-42_ on OWLS surfaces functionalised with both linear and branched KLVFF (Figure 6A,B). In particular, Aβ_1-42_ binding to the linear KLVFF showed a broader peak with an elution process characterised by a slower and irregular decay of the signals. Consistently, the OWLS-driven transformation of the binding data into mass values (ng/cm^2^) showed less chip surface functionalisation by the linear KLVFF peptides (Figure 7A) whereby a slower process of molecular adsorption reached a peak of 250 ng/cm^2^ and a slower wash out of the non-covalently bound excess (Figure 7A, 83–104 min) yielding ca 125 ng/cm^2^ covalently-bound peptide mass, whilst in the case of the functionalisation of the OWLS chip surface by Rgen3K(KLVFF)_16_, faster, more efficient covalent binding was observed after 74 min followed by a rapid removal (ca 1 min) of the unbound excess to yield a mass level of bound-branched peptides at level similar to that of the linear molecule (ca 125 ng/cm^2^) (Figure 7B). The similar level of coupled mass led to significantly different Aβ_1-42,_ binding profiles. Following injection, the AD biomarker molecules were captured by the chip surface functionalised with the linear aptamer at a slower rate and reached lower peaks of mass interaction and the slower removal of unbound excess molecules than those captured by the surface functionalised with Rgen3K(KLVFF)_16_ (Figure 7A,B). The resulting adsorbed Aβ_1-42,_ bound mass slowly increased from 125 to 387 ng/cm^2^ in the case of surfaces functionalised with KLVFF. The weakly bound excess mass being slowly removed to yield 301 ng/cm^2^ of stably bound Aβ_1-42_. Instead, Rgen3K(KLVFF)_16_ -modified chip surfaces showed a sharp and fast increase from ca 100 ng/cm^2^ 505 ng/cm^2^ in less than 2 min, followed by a fast and more efficient washing up of the unbound species in ca 3 min, thereby yielding a reliably measured Aβ_1-42_-detected mass of 250 ng/cm^2^. In other words, the dissociation of excessive bound Aβ_1-42_ observed in the case of chip surfaces functionalised with the linear peptide showed prolonged elution times highlighting less binding specificity and the likelihood of Aβ_1-42_ aggregate formation rolling and remaining adsorbed on the surface (Figure 7A, 110–130 min curve). Instead, in the case of chips functionalised with Rgen3K(KLVFF)_16_, the sharper and higher peak was followed by a very rapid decline to stable value (Figure 7B, 90–100 min) indicating a more specific and stable interaction, hence giving higher and more reliable measurements. No significant stable binding was observed when non-functionalised OWLS chip surfaces were used (data not shown).

## 4. Discussion

In agreement with previous studies [20], the present work shows that the KLVFF sequence is not only able to inhibit the overall process of Aβ_1-42_ self-aggregation but also to promote the disaggregation of formed fibrils upon 7 days. After 7 days’ incubation, the Aβ_1-42_ fibrils were observed to be shorter in the linear KLVFF-treated samples with a higher tendency to rearrange into amorphous aggregates when mixed with Rgen3K(KLVFF)_16_ but not with the hyperbranched peptide presenting the scrambled sequence VFLKF; i.e., Rgen3K(VFLKF)_16_ (Figure 5A). These results were also confirmed by the quantitative data shown in Figure 5B. The linear KLVFF was found to be able to decrease the intensity in *A*_540nm_ associated with Aβ_1-42_ fibrils formation resulting in a decrease of absorbance from 0.344 (Day1) to 0.299 within 7 days incubation when compared with Aβ_1-42_ control [Day1 = 0.355 *A*_540nm_; Day7 = 0.642 *A*_540nm_]. The more effective inhibition of the Aβ_1-42_ fibril aggregation process was shown by Rgen3K(KLVFF)_16_, which caused a drastic fibril breakdown already at 1 day [A_540nm_ = 0.276]. At day 7, these values were found to decrease further [0.182 *A*_540nm_] and to be sequence specific due to the inability of the scrambled Rgen3K(VFLKF)_16_ to prevent fibril self-aggregation [Day1 = 0.354 *A*_540nm_; Day7 = 0.577 *A*_540nm_]. Although not relevant to this study, the choice of performing the experiments at body temperature provides indication about the efficient inhibitory effect of the branched blocker in future therapeutic applications.

The aggregation of Aβ_1-42_ monomers into fibrils is currently considered the key pathogenic event in the onset of AD [21]. During pathogenic conditions, the HHQK motifs of Aβ_1-42_ are reported to be the main region involved in this process. These initially induce changes in the Aβ_1-42_ secondary structure by affecting the β-sheet-rich structure [22] and then lead individual monomers to interact with each other and form fibrils of distinct length [23]. This formation is triggered by intermolecular interactions between the hydrophobic regions of the Aβ_1-42_ [24] and especially those located at the residues 16–20 of Aβ_1-42_ [25]. Based on these results, KLVFF linear peptides have become one of the main lines of research to develop efficacious therapeutics for the early treatment of AD Aβ_1-42_ [26,27]. In this work, the ability of KLVFF linear peptides to bind to the Aβ_1-42_ and prevent their polymerisation into fibrils was demonstrated to increase once this sequence was integrated with the sixteen uppermost molecular ends of a hyperbranched poly(ɛ-lysine) bearing three branching generations. The relative hydrophobic nature of the KLVFF did not affect the synthesis of a more complex branched peptide; both Rgen3K(KLVFF)_16_ and KLVFF were synthesised at a high degree of purity and yield. The OWLS experiments showed that the more complex branched structure not only did not prevent the interaction with Aβ_1-42_ monomers but in fact improved it in terms of rapidity and stability of binding. Indeed, after the functionalisation of the waveguide surface with Rgen3K(KLVFF)_16_, the peak positions of both TE and TM gradually increased as peptides attached to the surface. The shift to elevated levels was in the accepted range of 0.005 and 0.01 incoupling angle shifts and reflected changes that in the case of branched KLVFF were significantly larger than those of the respective linear one. This was linked to the larger size of the hyperbranched molecule (≈100 times than KLVFF), which led Rgen3K(KLVFF)_16_ to homogeneously cover the whole waveguide surface. After the copious and protracted rinsing of the OWLS chip surface by deionised water, the peak position of both KLVFF and Rgen3K(KLVFF)_16_ did not return to their background levels indicating that both linear and branched KLVFF had been immobilised onto the chip surface. The concomitant changes in TE and TM of both Aβ_1-42_ inhibitors resulted in a further increase in surface mass as manifested after the injection of Aβ_1-42_. Moreover, nearly no decrease in sensor response was observed after three repetitive measurements, highlighting the specificity of the direct-binding affinity between Aβ_1-42_ with their inhibitors. However, when an Aβ_1-42_ solution was injected into the OWLS sensor chip functionalised with Rgen3K(KLVFF)_16_, this type of branched blocker demonstrated its ability not only to interact with Aβ_1-42_ but also to enhance its binding stability. The higher number of KLVFF motifs presented at the chip surface rather than the dendritic structure itself is thought to be the cause of this effect. Previous studies have demonstrated that the hydrophobic interactions existing between KLVFF and Aβ_1-42_ on residues 16-20 principally increased the binding capabilities of KLVFF by slowing the oligomerisation of Aβ_1-42_ while stabilising their monomer state [28]. Chafekar et al. (2007) reported the potential of 3^rd^-generation amine-terminated poly(amido amine) (PAMAM) dendrimers modified with KLVFF to control both Aβ_1-42_ aggregation and fibril formation in a well-defined manner [29]. This was mainly attributed to their relatively high hydrophobicity when compared with the effects of the linear KLVFF. Likewise, in this study, Rgen3K(KLVFF)_16_ was shown to be able to delay the formation of Aβ_1-42_ fibrils likely through the inhibition of the formation of β-sheet structures, which are known to be responsible for the development of more stable amyloid fibrils [30]. In addition to its Aβ_1-42_ fibril inhibitory effect, the KLVFF sequence has also been demonstrated to disrupt the formation of fibrils already formed [31]; in this work Rgen3K(KLVFF)_16_ was found to both enhance and protract the Aβ_1-42_ fibril disruptive properties of KLVFF. At day 1, a reduction in *A*_540nm_ compared the control and linear peptide indicated that fibril disassembly occurred already at the early stage of their development to be reduced even more after 7 days. The formation of smaller and immature fibrils that assembled in amorphous aggregates rather than into fibrils of different length and shape as those observed in Aβ_1-42_ -treated KLVFF samples is likely due to the combination of more hydrophobic domains and steric properties dictated by the branched structure of Rgen3K(KLVFF)_16_. However, it is worth to highlight that these inhibitory effects were principally triggered by the KLVFF sequence rather than by the dendritic structures themselves as both demonstrated in this study by the lack of inhibitory effect of the scramble branched sequence and confirmed by others [32]. Indeed, other studies have shown the lack of inhibitory properties in non-modified hyperbranched polyamine dendrimers to prevent the aggregation of amyloid fibrils [33] even if minor benefits were observed at increasing branching generation (e.g., 4th generation) [34].

This enhanced disaggregation effect of the Rgen3K(KLVFF)_16_ on Aβ_1-42_ fibril aggregation also improved the efficiency of OWLS detection of Aβ_1-42_ by reducing the artifact caused by the rolling and aspecific adsorption of both Aβ_1-42_ monomers and their aggregates on the chip surface. This seemed to be the case when the chip surface was functionalised with the linear KLVFF sequence. Indeed, sharp and higher mass increase and washing out of excess were observed in the former case, while slower binding and elution kinetics as well as an inefficient elution of excess mass were observed in the latter case. These differences in the binding properties could negatively affect the sensitivity and sensibility of the sensor in a clinical setup where body fluids, heterogeneous in composition, will inevitably lead to the competitive non-specific binding of other molecular species such as albumin [35]. The effect of the non-specific adsorption of other molecular species on the OWLS sensitivity and specificity was not verified in this study. Competitive studies with other serum proteins such as albumin that are known to bind most of the material surfaces should also be performed to validate further the OWLS AD diagnostic potential. However, this would require a dedicated study where concentrations and salt conditions as well as the use of human serum samples should be considered. Additionally, ELISA kits offer the opportunity to test multiple samples at the same time, a feature not currently available to OWLS sensors. However, it is worth highlighting that the widely accepted level of specificity of KLVFF makes their use comparable with that of ELISA methods based on more expensive monoclonal antibodies and that the OWLS sensitivity (nanogram/cm^2^) and speed of completion (few minutes of OWLS measurements against hours for ELISA tests) offer potential advantages in the accurate monitoring of disease onset and progression.

## 5. Conclusions

In this study, a hyperbranched molecular structure of the Aβ_1-42_ fibril inhibitor KLVFF, the Rgen3K(KLVFF)_16_, has been designed to increase the advantages of using this peptide in AD-specific in vitro diagnostics. When integrated in an OWLS system, they conferred specificity to the sensor, providing reliable results in the nanogram scale in no longer than 5 min. At the same time, the present work has demonstrated the manufacturing feasibility of the chip functionalisation process that can be obtained through the fast and scaled-up synthesis of very pure peptides as well as through an inexpensive method of chip functionalisation lasting less than 10 min. Therefore, the data show the potential of this sensing system to enable the early detection of the onset of Alzheimer’s Disease and monitor its progression thus enabling a timely and patient-tailored treatment.

## Data Availability

Raw data of the present work can be found in the University of Brighton public data repository.
